# Computational Studies on Sirtuins from *Trypanosoma cruzi*: Structures, Conformations and Interactions with Phytochemicals

**DOI:** 10.1371/journal.pntd.0002689

**Published:** 2014-02-13

**Authors:** Lionel Sacconnay, Melissa Angleviel, Giuseppe Marco Randazzo, Marcos Marçal Ferreira Queiroz, Emerson Ferreira Queiroz, Jean-Luc Wolfender, Pierre-Alain Carrupt, Alessandra Nurisso

**Affiliations:** School of Pharmaceutical Sciences, University of Geneva, University of Lausanne, Geneva, Switzerland; University of Alabama in Huntsville, United States of America

## Abstract

**Background:**

The silent-information regulator 2 proteins, otherwise called sirtuins, are currently considered as emerging anti-parasitic targets. Nicotinamide, a pan-sirtuin inhibitor, is known to cause kinetoplast alterations and the arrested growth of *T. cruzi*, the protozoan responsible for Chagas disease. These observations suggested that sirtuins from this parasite (TcSir2rp1 and TcSir2rp3) could play an important role in the regulation of the parasitic cell cycle. Thus, their inhibition could be exploited for the development of novel anti-trypanosomal compounds.

**Methods:**

Homology modeling was used to determine the three-dimensional features of the sirtuin TcSir2rp1 from *T. cruzi*. The apo-form of human SIRT2 and the same structure solved in complex with its co-substrate NAD^+^ allowed the modeling of TcSir2rp1 in the open and closed conformational states. Molecular docking studies were then carried out. A library composed of fifty natural and diverse compounds that are known to be active against this parasite, was established based on the literature and virtually screened against TcSir2rp1 and TcSir2rp3, which was previously modeled by our group.

**Results:**

In this study, two conformational states of TcSir2rp1 were described for the first time. The molecular docking results of compounds capable of binding sirtuins proved to be meaningful when the closed conformation of the protein was taken into account for calculations. This specific conformation was then used for the virtual screening of antritrypanosomal phytochemicals against TcSir2rp1 and TcSir2rp3. The calculations identified a limited number of scaffolds extracted from *Vismia orientalis*, *Cussonia zimmermannii*, *Amomum aculeatum* and *Anacardium occidentale* that potentially interact with both proteins.

**Conclusions:**

The study provided reliable models for future structure-based drug design projects concerning sirtuins from *T. cruzi*. Molecular docking studies highlighted not only the advantages of performing *in silico* interaction studies on their closed conformations but they also suggested the potential mechanism of action of four phytochemicals known for their anti-trypanosomal activity *in vitro*.

## Author Summary

*T. cruzi* is a protozoan pathogen responsible for Chagas disease. Current therapies rely only on a very small number of drugs, most of which are inadequate because of their severe host toxicity or because of their susceptibility to drug-resistance mechanisms. To determine efficient therapeutic alternatives, the identification of new biotargets and detailed knowledge of their structures are essential. Sirtuins from *T. cruzi* have been recently considered as promising targets for the development of new treatments for Chagas disease. Inhibition of their activity has been shown to significantly interfere with the life cycle of the parasite. *T. cruzi* possesses genes encoding two sirtuin-like proteins, TcSIR2rp1 and TcSIR2rp3. The structures of these enzymes were theoretically elucidated in this work, which also focused on the impact of their possible conformational states on computational interaction studies. A small library of phytochemicals that are active against the parasite was built and screened against the most meaningful conformations, identifying a restricted number of scaffolds that potentially interact with the modeled proteins. For these hits, a mechanism of action related to interactions with sirtuins was proposed.

## Introduction

Chagas disease is caused by a flagellated protozoan belonging to the *Trypanosomatidae* family of the *kinetoplastida* order. This disease which is mainly localized in Central and South America, affects approximately 8 million people, causing near 14,000 deaths worldwide [1] and leading to serious medical complications such as fatal damage to the heart muscles (cardiomyopathy), central nervous system, and digestive tract (megacolon, megaesophagus) consequently resulting in death [2], [3]. Unfortunately, the two drugs used for the treatment of Chagas disease (Benznidazole and Nifurtimoz), have limited efficacy and have been associated with numerous adverse side effects. Moreover, the pathogen seems to be able to develop resistance against these treatments [4], [5]. Thus, there is an urgent need to find new biotargets for the development of compounds with low toxicity and good efficacy against this parasite [6].

**Figure 1:**
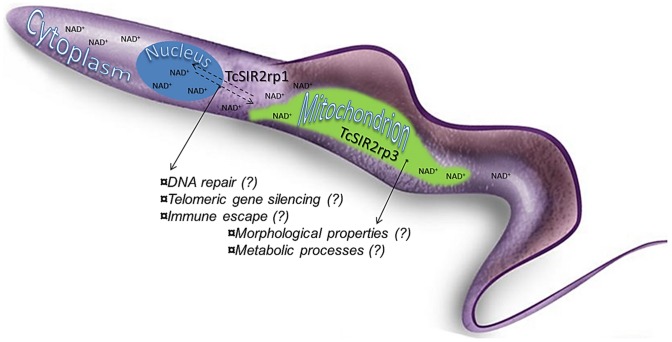
Hypothetical roles of NAD^+^ dependent deacetylases (sirtuin family) in the *T. cruzi* parasite.

The silent-information regulator 2 (SIR2) proteins are currently considered as emerging anti-parasitic targets because of their nicotinamide adenine dinucleotide NAD^+^-dependent deacetylase activity on histones and other cellular substrates. It has been demonstrated, for example, that sirtuins from *Plasmodium* species are involved in the regulation of the telomere-associated *var* gene family members that encode for proteins responsible of host immune evasion [7], [8]. *Leishmania* sirtuins were found to be implicated in delaying apoptosis and providing protection from host immune responses [9], [10], [11]. Moreover, their species specific inhibition by bisnaphthalimidopropyl (BNIP) derivatives has exhibited robust anti-leishmanial activity [12]. Whereas TbSir2rp2 and TbSir2rp3 from *T. brucei* do not impact on parasite growth and differentiation *in vitro*, TbSir2rp1 has been shown to participate in DNA repair through the modification of H2A and H2B histones [13], [14]. For an extensive review on this topic, please refer to [15], [16]. In *T. cruzi*, two isoforms from the SIR2 family have been identified: TcSIR2rp1 and TcSIR2rp3 (Figure 1) [17]. It was proven *in cellulo* that their inhibition by the well-known sirtuin inhibitor nicotinamide can cause morphologic alterations and an inhibitory growth of this parasite, suggesting a potential use of TcSIR2 proteins for the development of new drugs against Chagas disease [18].

From a structural point of view, sirtuins are formed by a large Rossmann fold domain and by a small domain composed of a helical and a zinc-binding module. Their catalytic pocket is conserved, from bacteria to mammals [19], [20] and it is formed by three sub-pockets that accommodate the co-substrate NAD^+^: the A pocket, where the adenine-ribose moiety binds; the B pocket, where the nicotinamide-ribose moiety binds; and the C pocket, where nicotinamide is supposed to bind [21]. Recently, crystallography identified two possible conformational states for sirtuins: an open or non-productive conformation (apo-form) and a closed or productive conformation (bound-form). The two conformers, experimentally observed in the human sirtuin SIRT2, differ at the catalytic pocket in terms of shape and ligand accessibility [21], [22]. By considering this very recent structural information, two conformers of TcSIR2rp1 have been built by homology modeling. A molecular docking approach was then used not only for evaluating the quality of the modeled proteins but also for understanding the impact of the two conformational states on the final docking results. It is important to note that the majority of molecular docking calculations involving sirtuins reported in the literature have been carried out using only the non-productive conformation of sirtuins [23], [24], [25].

Finally, because nature is a rich source of anti-trypanosomal agents [26], [27], a database of phytochemicals with a known inhibitory activity *in vitro* against *T. cruzi* has been collected from the literature. Computational interaction studies between these compounds and sirtuins in the more meaningful modeled conformation was conducted to identify a potential sirtuin-related trypanocidal activity.

## Materials and Methods

### Homology modeling

Homology modeling was performed by using the Molecular Operating Environment MOE software package (MOE 2012.10; Chemical Computing Group, Montreal, Canada). The primary sequence of SIR2rp1 from *T. cruzi* (TcSIR2rp1, sequence ID: Q4DP02) was retrieved from the Universal Protein Resource database and used as a target for the homology modeling. To identify a template structure, the target sequence was submitted to a PSI-BLAST search (http://blast.ncbi.nlm.nih.gov/) against the Protein Data Bank PDB (www.rcsb.org/) using the BLOSUM62 matrix with an E-value cutoff of 10. The human cytosolic SIRT2 protein (hSIRT2), having ∼35% sequence identity with the target, was selected as a template for the current project. Two conformations of hSIRT2 were found in the PDB: a non-productive conformation (PDB ID: 1J8F), that corresponds to the apo-hSIRT2 [21], and a productive one (PDB ID: 3ZGV) [22], in which hSIRT2 binds adenosine-5-diphophoribose A5dPR. Both proteins were used in the study. Thus, TcSIR2rp1 was modeled in the two possible conformational states by following the same strategy. The target-template sequence alignment was performed using MOE's multiple sequence alignment algorithm [28], [29] and then refined manually. Three-dimensional model building was then carried out using the MOE homology program [30] based on a segment matching procedure [31]. Ten intermediate models of TcSIR2rp1 in both non-productive and productive forms were generated and successively minimized by using the Tripos force field [32] in Sybyl-×2.0 (TRIPOS Inc., St. Louis, MO). The sequence from Trp93 to His97 of the parasitic productive form was modeled based on the productive human SIRT1 structure (PDB ID: 4I5I) due to a lack of structural information of this specific loop in hSIRT2 [33]. Gasteiger-Huckel charges were assigned to the proteins before starting the minimization step that was conducted using the Tripos force field [32] with 700 cycles of the Powell algorithm. Finally, the stereochemical quality of the models was assessed using Ramachandran plot analysis included in the PROCHECK validation package (http://nihserver.mbi.ucla.edu/SAVES/). The models with the best stereochemical quality were then selected for the interaction studies described in the next section.

### Molecular docking

The crystal structure of hSIRT2 co-crystallized with the co-substrate A5dPR (PDB ID: 3ZGV) was taken into account not only as a template for the homology modeling but also for validating the docking methodology. This structure was prepared for docking in Sybyl-×2.0 (TRIPOS Inc., St. Louis, MO) by removing the crystallized water molecules, by adding the missing hydrogen atoms and by extracting the ligand. The re-docking was then conducted using GOLD version 5.1 (CCDC, Cambridge, UK). The binding site was defined by a 12.5 Å radius sphere around the Ala85 residue and 100 docking solutions were generated by using 100,000 GOLD Genetic Algorithm iterations (Preset option). Docking poses were evaluated and ranked according to the PLP score. Root-mean-square-deviation RMSD values between the docking solutions and the crystallized ligand of reference were calculated to evaluate docking performance. The homology models were then superimposed on the respective productive (PDB ID: 3ZGV) and non-productive (PDB ID: 1J8F) hSIRT2 conformations using the nicotinamide recognition sequence (TQNXD motif) as a reference for superimposition [16]. Further docking studies of NAD^+^ (co-substrate), nicotinamide (pan-sirtuin inhibitor) and AGK2 (selective hSIRT2 inhibitor) were carried out on the non-productive and productive protein forms of *T. cruzi* sirtuins by applying the methodology described above [18], [34], [35]. The binding site of the parasitic targets was defined by taking Ala38 into account as a reference for NAD^+^ and AGK2 docking whereas, for nicotinamide, the binding site was defined by taking into account an 8 Å radius sphere around Asp125. This correction was made to avoid false positive results due to the small size of this compound with respect to the sirtuin pocket. Clustering analysis was finally performed using the RMSD analysis tool implemented in GOLD version 5.1.

### Library of trypanocidal natural compounds: Construction and diversity

A library of fifty compounds with *in vivo* anti *T. cruzi* activity was created by collecting data from the literature (Table S1). Iridoids & Terpenes (11 compounds), Phenolics (31 compounds) and Alkaloids (9 compounds) characterize the library. 3D structures were generated by using Sybyl-×2.0 (TRIPOS Inc., St. Louis, MO), with particular regard to the stereochemistry and protonation states (pH = 7.4). To display the chemical variation of data, a heat map based on the Tanimoto similarity matrix of Morgan fingerprints [36], was generated using RDKit [37], SciPy [38] and Matplotlib [39] tools.

### *In silico* screening of trypanocidal natural compounds against sirtuins

The database was screened against the productive form of *T. cruzi* sirtuins (TcSIR2rp1, TcSIR2rp3 [40]) using the previously described protocol. The best-ranked forms according to their PLP score were taken into account for docking evaluation. The compounds with a positive score with respect to AGK2 (potent and selective class I sirtuin inhibitor [35]) and thiobarbiturate **6** (potent and selective class III sirtuin inhibitor [41]) were selected for further structural analyses.

## Results and Discussion

### Homology modeling of Sir2rp1 from *T. cruzi*: Non-productive and productive conformational states

**Figure 2:**
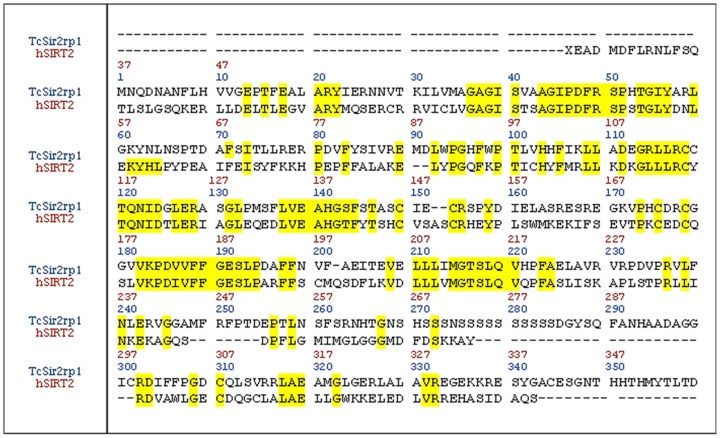
Sequence alignment between TcSir2rp1 and hSIRT2. Conserved amino acids are highlighted in yellow.

A rational template search was the first step for the construction of a reliable homology model of TcSIR2rp1. Through a BLAST-P search, the human histone deacetylase hSIRT2, with ∼35% of sequence identity, was determined to be the best template choice for TcSIR2rp1 modeling [21]. The primary sequence of the selected template was aligned with the respective target primary sequence (Figure 2). The alignment showed a high number of conserved residues (∼70%) in the three catalytic sub-pockets (Table S2).

As reported for hSIRT2, two possible conformational states in sirtuins could exist: a so-called non-productive conformation, characterized by the absence of ligands in the catalytic site [21], and a productive one, in which the protein is found in a bound state [22]. The main difference between the two conformations consists of a 25° rotation of the zinc-binding domain towards the Rossmann-fold domain. Moreover, a high flexibility of the zinc-binding domain was observed in the productive form (Figure S1). In this study, two conformations of TcSIR2rp1 were modeled, based on hSIRT2 structural information. After several energy minimization cycles, 98.3% and 98.6% of φ and ψ backbone dihedral angles from the non-productive and productive protein forms occupied the allowed regions of the Ramachandran plots whereas less than 1%, comprised of residues that are not part of the active site regions, occupied the disallowed ones (Figure S2).These percentages, together with the absence of clashes and anomalies in bond lengths and angles, emphasized the accuracy of the modeled proteins.

### A productive conformational state of TcSir2rp1 is necessary for reliable molecular docking studies

**Figure 3:**
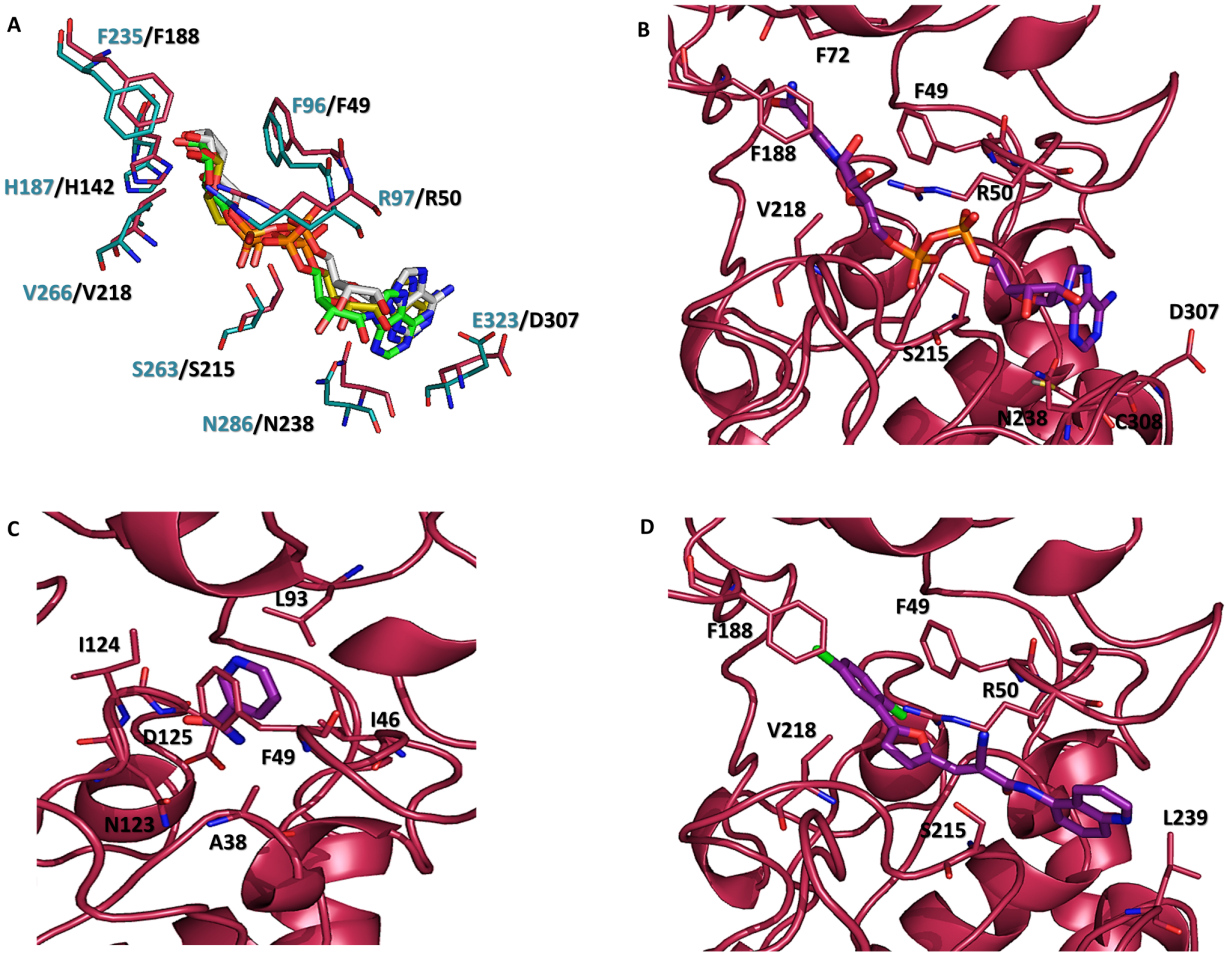
Molecular docking results in the productive pocket of TcSIR2rp1. (A) Superimposition of A5dPR crystal (yellow-capped sticks) and best-ranked docking poses in hSIRT2 (green-capped sticks, cyan ribbons) and TcSIR2rp1 (white-capped sticks, dark pink ribbons). (B) NAD^+^ best-ranked docking pose in the TcSIR2rp1 productive form (purple-capped sticks, dark pink ribbons). (C) Nicotinamide best-ranked docking pose in the TcSIR2rp1 productive form (purple -capped sticks, dark pink ribbons). (D) AGK2 best-ranked docking pose in the TcSIR2rp1 productive form (purple-capped sticks, dark pink ribbons).

In this project, a molecular docking approach was used to verify the quality of the modeled proteins. Moreover, the impact of the two modeled conformational states on the final docking results was also evaluated. To validate the docking methodology, a re-docking strategy was carried out using the structure of A5dPR co-crystallized with hSIRT2 (PDB ID: 3ZGV, productive conformation). The best-ranked docking solution according to the PLP score presented an RMSD value of 0.7 Å with respect to the crystallographic pose. Contacts with the protein were mainly polar, involving Arg97, Asn286, His187 and Glu323 residues [22]. Hydrophobic interactions with Phe235, Val266 and Phe96 were also present. When the same ligand was docked into the modeled productive TcSIR2rp1 binding site, a similar binding mode and interaction network were determined. Figure 3A shows the docking results, highlighting the successful reproduction of the crystallographic information *in silico*.

NAD^+^ was successively docked into the productive form of TcSir2rp1 (Table S3). The best-ranked solution, according to the PLP score, was evaluated using the crystallographic NAD^+^ pose in the hSIRT1 pocket as a reference [33]. The hydrogen bond network described by Zhao *et al.* was retrieved and it involves Ala38, Phe49, Arg50, Thr214, Ser215, Asn238, and Cys308 residues. Hydrophobic contacts stabilizing the nicotinamide ring were also observed (Figure 3B).

Nicotinamide was then docked into the productive pocket of TcSIR2rp1 [42]. In agreement with the crystallographic information from the *Thermotoga maritima*-nicotinamide Sir2 homolog complex (PDB ID: 1YC5) [43], the inhibitor occupied the C-site of the protein, forming hydrogen bonds with the Ile124 backbone and Asp125 lateral chain. ∏-stacking interactions with Phe50 were also observed along with hydrophobic contacts involving Ile124, Ala38 and Ile46 (Figure 3C).

AGK2 was finally docked into the productive binding site of TcSIR2rp1 (Figure 3D, Table S3). This compound was demonstrated to have a certain degree of selectivity towards class I sirtuins [44]. To date, no information about the potency of AGK2 trypanocidal activity is available. However, due to the high identity between the catalytic pocket of hSIRT2 and TcSIR2rp1 [24], a similar binding mode was assumed in the current study (Figure 3A, Table S2). It has been described that AGK2 in hSIRT2 can preferentially bind the C-pocket of this protein [35]. However, in the absence of crystallographic information, it can be supposed, by a structure-activity relationship, that AGK2 can assume an orientation similar to A5dPR, occupying the A and B pockets. Our docking results are seemingly in agreement with this hypothesis (Figure 3D, Figure S3).

**Figure 4:**
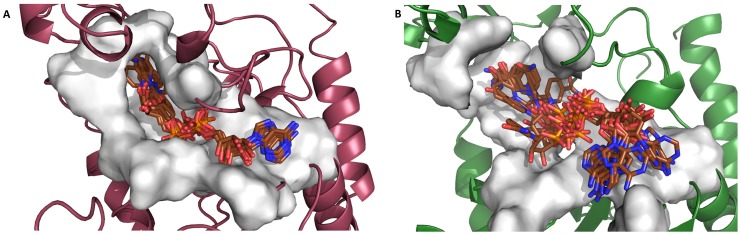
Superimposition of ten NAD^+^ docking poses in the TcSIR2rp1 productive (A) and non-productive (B) forms. NAD^+^ molecules are represented in brown-capped sticks. Protein structures are represented as ribbons and colored in dark pink and green representing the TcSIR2rp1 productive and non-productive form, respectively. Pocket surfaces were generated with MOE (MOE 2012.10; Chemical Computing Group, Montreal, Canada) and are colored in gray.

By following the same methodology, *in silico* interaction studies were also carried out on the non-productive form of TcSIR2rp1. The docking analysis revealed that the NAD^+^ best-ranked pose occupied a different position in the active site, compared to the hSIRT1 co-crystal [25], with the adenosine-ribose moiety pointing toward the solvent. A cluster analysis (cut-off of 2 Å) performed on the docking solutions (Table S4), indicated that 24% of the docking positions converged to this binding mode. Moreover, numerous and diverse docking clusters, related to the high number of conformations the ligand can assume in the wide pocket, were obtained (Figure 4A). Conversely, cluster analysis performed on the docking solutions from the productive form highlighted a more important convergence (46%) to the correct orientation of the co-substrate in the pocket (Figure 4B). In agreement with the previous findings, the docking of nicotinamide in the TcSIR2rp1 non-productive form showed that all docking poses occupied a non-crystallographic position in the pocket whereas all the docking solutions retrieved in the productive form matched the crystallographic orientation (Table S4, Figure S4). Finally, the best-ranked pose of AGK2 seemed, even in the TcSIR2rp1 non-productive form, able to adopt a position similar to the one retrieved in the productive active site. However, this specific solution belonged to a cluster populated by a small portion of docking solutions (16%) whereas, in the productive form, 27% of the docking poses converged to the expected one (Table S4, Figure S4). Nevertheless, the PLP scores obtained for all the ligands docked in the productive TcSIR2rp1 were higher if compared to the scores obtained in the non-productive form (Table S3). This observation can be explained by the lack of key interactions between ligands and the non-productive form of the enzyme that is unfavorable for ligand accommodation. By considering all these observations, the productive form of TcSIR2rp1, herein modeled for the first time, will be taken into account for the interaction studies described in the following section.

### Computational interaction studies identified a possible sirtuin-related mechanism of action of four trypanocidal natural compounds

*In silico* target-fishing approaches have been reported in the past to identify the possible mechanism of actions of anti-parasitic compounds [45], [46]. By following a similar strategy, with the aim of identifying a possible action related to sirtuin inhibition, fifty diverse anti-trypanosomal natural compounds were docked into the productive forms of sirtuins from *T. cruzi*: TcSIR2rp1 and TcSIR2rp3 [40] (Table S1). The latter has been recently modeled in a productive conformational state based on the *E. coli* CobB protein and *A. fulgidus* sirtuin in complex with NAD^+^ (PDB IDs: 1S5P, 1ICI). As qualitatively demonstrated by the color of the heat map reported in Figure S5, the compounds characterizing the small dataset used in this work were diverse in terms of chemical structure. The results of the screening are listed in Table 1. PLP scores were compared to AGK2 and thiobarbiturate 6, because these compounds are selective inhibitors of sirtuin class I [35] and class III [41], respectively. Four compounds, according to their positive score with respect to the score obtained for the reference compounds, were selected for further structural inspections as potential sirtuin modulators: anacardic acid derivative (Cmp5), aculeatin D (Cmp33), 16-acetoxy-11-hydroxyoctadeca-17-ene-12,14-diynylethanoate (Cmp1) and vismione D (Cmp50). GRID surfaces were then calculated in order to better understand the interactions with the proteins [47]. DRY, N1 and O probes were used to describe the hydrophobic, electron-donor and electron-acceptor properties of the pockets. Interestingly, the analysis of the docking poses suggested that each ligand is a competitive inhibitor for NAD^+^ fixation, being able to occupy the NAD^+^ pocket of the proteins.

**Table 1:** PLP scores for the anti-trypanosomal compounds docked into *Tc*SIR2rp1 and *Tc*SIR2rp3 productive forms.

	Compound (Cmp) Names	TcSir2rp1	TcSIR2rp3	hSIRT2	hSIRT5	Ref.
	AGK2	71.2		73.3	-	[35]
	Thiobarbiturate 6		72.3	-	58.8	[41]
**1**	**16-acetoxy-11-hydroxyoctadeca-17-ene-12,14-diynylethanoate**	**77.0**	**80.1**	**98.9**	**89.5**	[**50**]
2	6′-O-acetyldiderroside	59.1	57.1	-	-	[51]
3	Ambigol A	54.1	62.7	-	-	[52]
4	Ambigol C	63.5	74.6	-	-	[52]
**5**	**Anacardic acid (8E, 11E)**	**80.7**	**83.1**	**92.0**	**96.6**	[**53**]
6	Ancistectorine A2	42.0	43.8	-	-	[54]
7	Ancistrogriffine C	−2.6	52.8	-	-	[55]
8	Ancistrogriffithine A	16.0	45.5	-	-	[55]
9	Angoroside C	59.2	69.0	-	-	[56]
10	Caaverine	45.9	53.7	-	-	[57]
11	Caffeic acid	39.5	53.5	-	-	[58]
12	Chaetoxanthone A	36.8	58.9	-	-	[59]
13	Chaetoxanthone B	37.0	55.4	-	-	[59]
14	Chaetoxanthone C	45.8	47.5	-	-	[59]
15	Cissampelofavone	45.3	68.6	-	-	[60]
16	7-deacetyl-gedunin	4.7	55.1	-	-	[61]
17	Demethyl-praecanson A	69.8	63.4	-	-	[62]
18	2,2-dimethyl-6-carboxyethenyl-2H-1-benzopyrane	55.1	62.9	-	-	[63]
19	2,2-dimethyl-6-carboxyethenyl-8-prenyl2H-1-benzopyrane	72.9	68.0	-	-	[63]
20	6,6-dimethyl-2-methoxy-6H-benzo[c]chromen-9-yl)methanol	49.0	63.5	-	-	[64]
21	Ent-kaurenoic acid	40.3	54.0	-	-	[65]
22	Ent-naringeninyl-(I-3α,II-8)-4′-O-methylnaringenin	25.8	30.2	-	-	[66]
23	Gallocatechin gallate	65.8	79.0	-	-	[67]
24	γ-fagarine	35.6	54.6	-	-	[68]
25	Garciniaxanthone B	43.7	45.8	-	-	[69]
26	Geranylgeraniol	67.6	76.4	-	-	[70]
27	Garcilivin A	25.9	37.3	-	-	[66]
28	Haemanthamine	44.6	58.6	-	-	[71]
29	Helenalin	40.6	49.2	-	-	[72]
30	Hydroxyanthecotulide	58.3	66.2	-	-	[73]
31	3-hydroxydaidzein	52.7	58.7	-	-	[74]
32	Rel-(7R,8R)-8-[(E)-3-hydroxy-3-methyl-1-butenyl]-4,8-dimethoxy-5,6,7,8-tetrahydrofuro[2,3b]quinoline-7-yl acetate	65.0	55.8	-	-	[75]
**33**	**Aculeatin D**	**79.7**	**90.3**	**85.7**	**92.7**	[**76**]
34	Ivalin	45.7	55.5	-	-	[72]
35	Komarovinone A	57.8	42.7	-	-	[77]
36	Luteolin	61.7	66.9	-	-	[58]
37	Isosakuranetin	36.8	53.1	-	-	[78]
38	Methylpluviatolide	56.8	84.4	-	-	[79]
39	4′-O-demethylancistrocladinium A	36.9	56.3	-	-	[80]
40	Parthenolide	43.1	47.0	-	-	[81]
41	4′-O-demethylknipholone-4′-O-β-d-glucopyranoside	25.8	53.1	-	-	[82]
42	Piperine	65.6	68.3	-	-	[83]
43	3-prenyl-4-hydroxycinnamic acid	55.2	67.4	-	-	[63]
44	3,5-piprenyl-4-hydroxycinnamic acid	69.1	70.6	-	-	[63]
45	Sarachine	36.0	60.7	-	-	[84]
46	Sophoraflavone G	54.7	85.0	-	-	[85]
47	Tiliroside	48.9	95.3	-	-	[53]
48	Usnic acid (R)	38.6	53.2	-	-	[86]
49	Usnic acid (S)	36.7	65.6	-	-	[87]
**50**	**Vismione D**	**77.1**	**77.7**	**91.4**	**81.3**	[**88**]

The compounds selected from the virtual screening for structural inspections are reported in bold.

**Figure 5:**
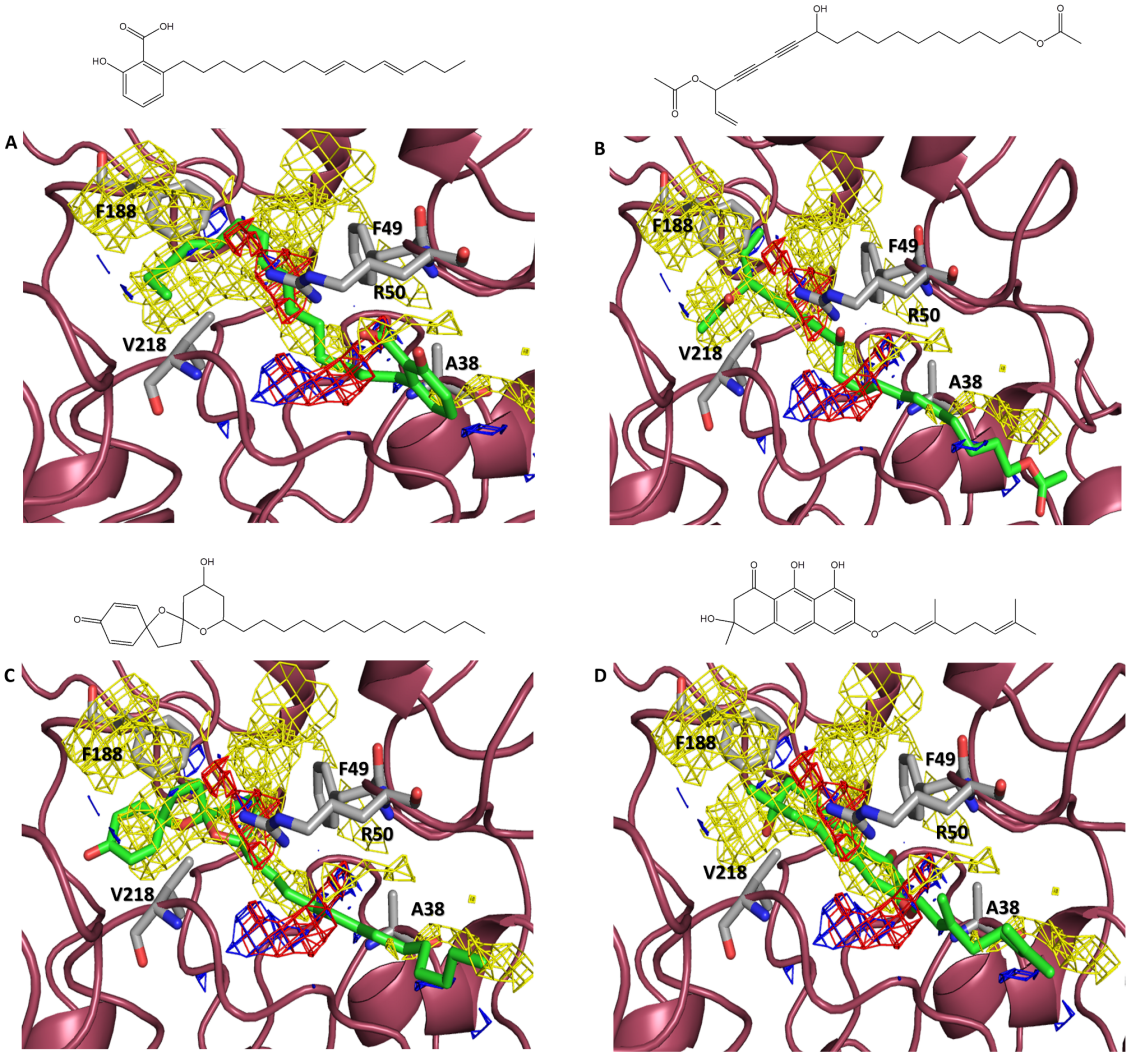
Molecular docking results from the virtual screening of the productive conformational state of TcSIR2rp1. (A) Anacardic acid best-ranked docking pose in the TcSIR2rp1 productive form. (B) Aculeatin D best-ranked docking pose in the TcSIR2rp1 productive form. (C) 16-acetoxy-11-hydroxyoctadeca-17-ene-12,14-diynylethanoate best-ranked docking pose in the TcSIR2rp1 productive form. (D) Vismione D best-ranked docking pose in the TcSIR2rp1 productive form. Protein structures are represented as dark pink ribbons. Amino acids participating in protein-ligand interactions are indicated bylight gray sticks. Ligands are represented in capped sticks and are colored in orange. GRID surface are also reported in the active site pockets and are colored as yellow, blue and red to highlight the hydrophobic, electron-donor and electron-acceptor properties, respectively.

Considering the PLP score, the anarcadic acid derivative (Cmp5) obtained from the cashew nut shell liquid exhibited the best overall docking results in both parasitic sirtuins. The best-ranked pose of this compound in the TcSIR2rp1 pocket formed hydrogen bonds between the carboxylic group of the ligand and the side chain of Arg50. Moreover, additional hydrophobic interactions with Ala38, Phe49, Phe188 and Val218 were observed (Figure 5A). Surprisingly, in TcSIR2rp3, the best-ranked pose exhibited a shift of 180° in the binding site with the poly-carbonated chain pointing toward the A pocket. A hydrogen bond between the polar head of the compound and Arg60, which is known to interact with the succinyl/malonyl group of the endogenous substrate and also to be responsible for nicotinamide resistance in sirtuin class III proteins, was highlighted [15], [48]. Hydrophobic interactions between the lipophilic chain and Ala14, Phe24, Phe157 and Val186 residues of pocket B and C were also detected. The second best-ranked pose matched the one observed in TcSIR2rp1, suggesting two possible binding modes in TcSIR2rp3 (Figure 5A, S6A, S7). All these observations correlate well with the GRID surfaces calculated in the protein pockets, especially with the DRY surfaces, indicating the main role of hydrophobic interactions in the binding.

Aculeatin D (Cmp33) extracted from the rhizomes of *Amomum aculeatum* was characterized by PLP scores of 79.7 and 90.3 in the TcSIR2rp1 and TcSIR2rp3 productive forms, respectively. As in the previous case, hydrophobic-driven interactions with the sirtuin pockets, also highlighted by the DRY probe, were observed (Figure 5B). In addition, for TcSIR2rp3, hydrogen bonds with Val97, Arg60 and His113 were reported (Figure S6B), explaining the higher score observed for this specific isoform.

16-acetoxy-11-hydroxyoctadeca-17-ene-12,14-diynylethanoate (Cmp1) extracted from the root bark of *Cussonia zimmermannii* formed similar interactions with both proteins. Hydrogen bonds between the hydroxyl and carbonyl group of the polar head with, respectively, Arg50 and Cys308 of TcSIR2rp1 and Asn185 and Lys224 of TcSIR2rp3 were observed. Hydrophobic interactions with Ala38, Phe49, Phe188 and Val218 of TcSIR2rp1 and Ala14, Phe25, Phe157 and Val186 of TcSIR2rp3 were also detected (Figure 5C, FigureS6C). Again, GRID surface analysis highlighted a good match between DRY surfaces and the hydrophobic region of the potential inhibitor.

Vismione D (Cmp50) isolated from the plant *Vismia orientalis Engl*. (Guttiferae or Clusiaceae) was the last compound that was selected from the screening, according to the PLP score. Hydrogen bonds with Ala38 (TcSIR2rp1) and Ala14 (TcSIR2rp3) backbones were detected. Moreover, van der Waals contacts with the hydrophobic amino acids characterizing the pocket were retrieved in both parasitic proteins (Figure 5D, Figure S6D).

These results suggested that the possible mechanism of action of these four natural compounds, that are known to have an inhibitory activity against *T. cruzi*, could be related to interactions with sirtuins. Indeed, the compounds identified by molecular docking are flexible molecules that would likely bind many active sites such as other NAD^+^ dependent enzymes. Additional docking studies against other enzymes (NAD^+^ dependent and others) would clarify this point. Moreover, no selectivity was found when the same computational approach was performed using the homologous human enzymes hSIRT2 and hSIRT5 (Figure S8). Unfortunately, these quite large lipophilic compounds, displaying no selectivity for parasite protein over host, do not appear to be very drug-like starting points. Please refer to the works of Kaur et al. and Sacconnay et al. for information about structural differences in the binding pockets that could be exploited for species-specific sirtuin inhibitor design [40], [24]. Nevertheless, in this study, several goals were reached: (i) the building (*in silico*) and the description of the three-dimensional structure of two conformational states of TcSIR2rp1 from *T. cruzi*; (ii) the evaluation of their impact on docking calculations, showing the advantages of performing computational interaction studies on the productive form; and (iii) the identification of a possible sirtuin-related mechanism of action of four natural trypanocidal compounds. Indeed, such results require experimental validation. The homology models of *T. cruzi* sirtuins are deposited in the Protein Model Database PMDB [49] with the following IDs: PM0079211 (*Tc*SIR2rp1, non-productive conformational state [40]), PM0079212 (*Tc*SIR2rp1, productive conformational state), and PM0078446 (*Tc*SIR2rp3, productive conformational state).

## Supporting Information

Figure S1 **Structural superimposition of the non-productive form (light pink ribbons) and productive form (purple ribbons) of TcSir2rp1.**
(PDF)

Figure S2 **Ramachandran plots of the non-productive (a) and productive (b) forms of Tcsir2rp1 homology models.**
(PDF)

Figure S3 **NAD^+^ (A), nicotinamide (B) and AGK2 (C) best-ranked docking poses in the hSIRT2 productive form.** Protein is represented in deep cyan cartoon whereas the amino acids involved in the interaction with the ligands are represented as capped sticks. NAD^+^, Nicotinamide and AGK2 are also represented as capped sticks and are colored in brown, orange and purple, respectively.
(PDF)

Figure S4 **Superposition of ten nicotinamide and AGK2 docking poses in TcSIR2rp1 productive (A–C) and non-productive (B–D) forms.** Nicotinamide and AGK2 are represented by orange and purple-capped sticks respectively. Backbones are represented with ribbons and are colored in dark pink and green, representing the TcSIR2rp1 productive and non-productive form respectively. Surfaces of the protein pockets are colored in gray.
(PDF)

Figure S5 **Heat map constructed from the fifty natural compounds of the library.** Colors in the heat map indicate the relative similarity (brown for high similarity and white for low similarity) of the molecules in the dataset.
(PDF)

Figure S6 **Molecular docking results in the productive pocket of TcSIR2rp3.** (A) Anacardic acid docking pose in the TcSIR2rp3 productive form. (B) Aculeatin D best-ranked docking pose in the TcSIR2rp3 productive form. (C) 16-acetoxy-11-hydroxyoctadeca-17-ene-12,14-diynylethanoate best-ranked docking pose in the TcSIR2rp3 productive form. (D) Vismione D best-ranked docking pose in the TcSIR2rp3 productive form. Protein structures are represented as light pink ribbons. Amino acids participating in protein-ligand interactions are highlighted by light gray sticks. Ligands are represented as capped sticks and are colored in orange. GRID surface are also reported in the active site pocket and are colored yellow, blue and red for hydrophobic, electro-donor and electro-acceptor properties, respectively.
(PDF)

Figure S7 **Best-ranked docking poses for the Anacardic acid derivative in TcSIR2rp3 (A) and hSIRT5 (B) productive forms.** Protein structures are represented in ribbons and colored in pink and light blue for TcSIR2rp3 and hSIRT5, respectively. Amino acids participating in protein-ligand interactions are represented in orange capped sticks. Ligand is represented in capped stick and colored in orange. GRID surfaces are also reported in the active site pockets and colored as yellow, blue and red for highlighting hydrophobic, electro-donor and electro-acceptor properties, respectively.
(PDF)

Figure S8 **Molecular docking results from the virtual screening in the productive conformational states of hSIRT2 and hSIRT5.** Anacardic acid best-ranked docking poses in hSIRT2 (A) and in hSIRT5 (B). Aculeatin D best-ranked docking poses in hSIRT2 (C) and in hSIRT5 (D). 16-acetoxy-11-hydroxyoctadeca-17-ene-12,14-diynylethanoate best-ranked docking poses in hSIRT2 (E) and in hSIRT5 (F). Vismione D best-ranked docking poses in hSIRT2 (G) and in hSIRT5 (H). Protein structures are represented as ribbons and are colored cyan and light blue for hSIRT2 and hSIRT5 respectively. Amino acids participating in protein-ligand interactions are represented as light gray sticks. Capped stick ligands are colored orange. GRID surfaces are also reported in the active site pockets and are colored yellow, blue and red to highlight hydrophobic, electron-donor and electron-acceptor properties, respectively.
(PDF)

Table S1 **Smile codes for the anti-** ***T. cruzi*** **compounds characterizing the natural product library.**
(PDF)

Table S2 **Summary of the residues characterizing the A–C pockets of TcSir2rp1 and hSIRT2.**
(PDF)

Table S3 **Best-ranked PLP docking scores of NAD^+^, nicotinamide and AGK2 ligands in the non-productive/productive TcSIR2rp1 conformations.**
(PDF)

Table S4 **Clustering analysis results (2 Å cut-off).** Red clusters contain the best-ranked poses according to the PLP score.
(PDF)
