# Supplementary figures and images for: Computational Studies on Sirtuins from Trypanosoma cruzi: Structures, Conformations and Interactions with Phytochemicals
Source: PLoS Negl Trop Dis. 2014 Feb 13;8(2):e2689. doi: 10.1371/journal.pntd.0002689 (PMC3923677; doi:10.1371/journal.pntd.0002689)

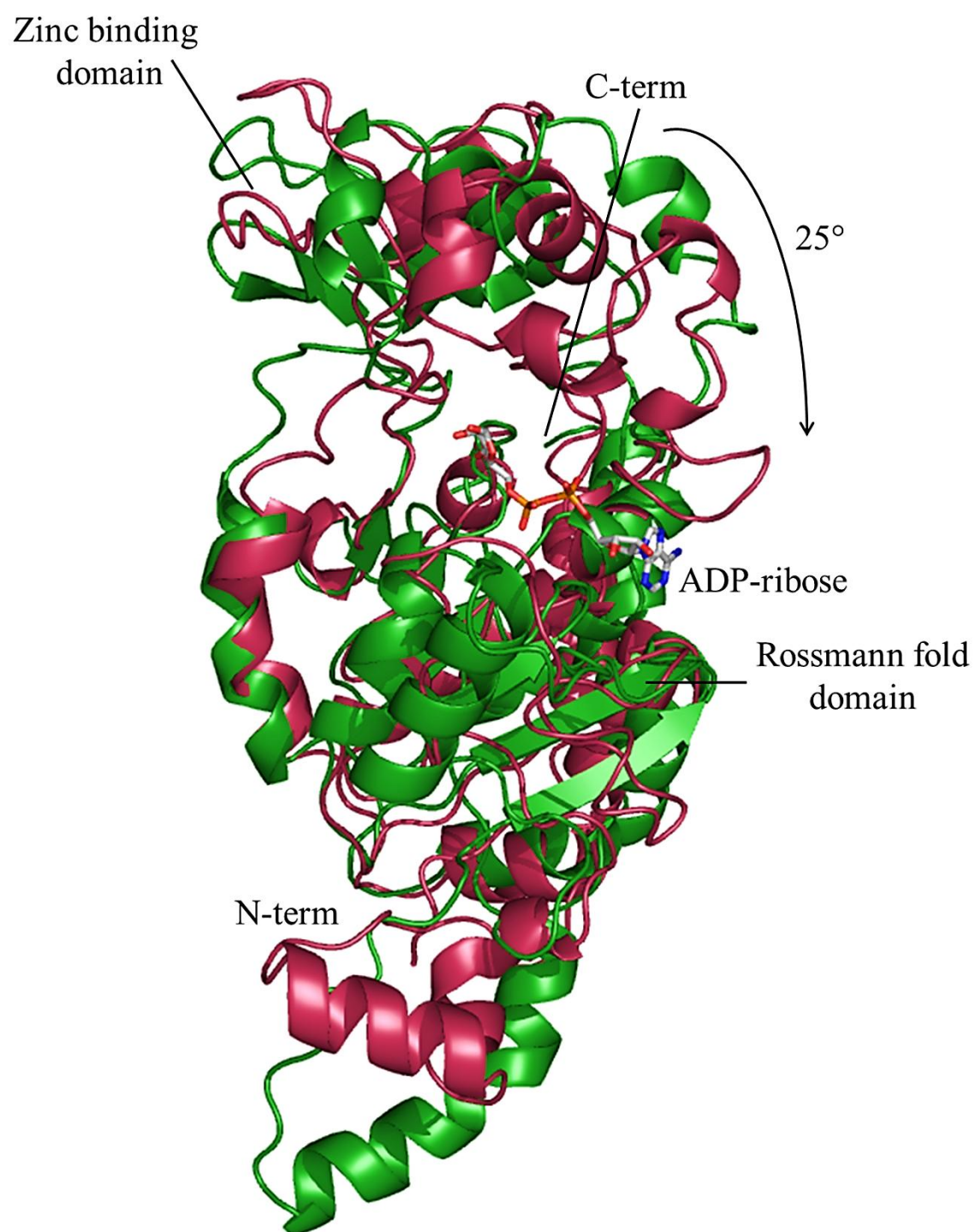

Supplement: Figure S1 — Structural superimposition of the non-productive form (light pink ribbons) and productive form (purple ribbons) of TcSir2rp1. (PDF) [file pntd.0002689.s001.pdf]

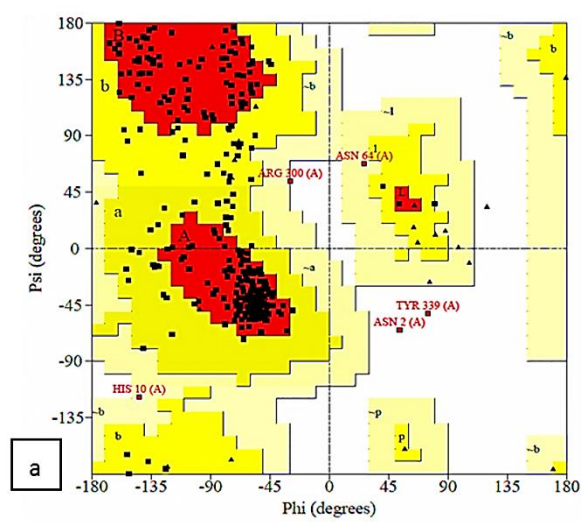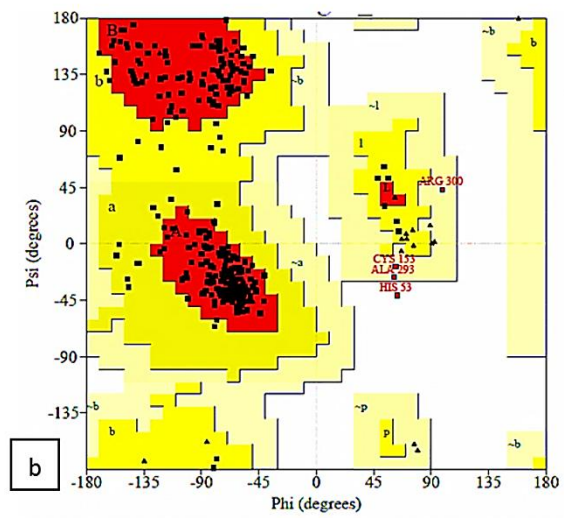

Supplement: Figure S2 — Ramachandran plots of the non-productive (a) and productive (b) forms of Tcsir2rp1 homology models. (PDF) [file pntd.0002689.s002.pdf]

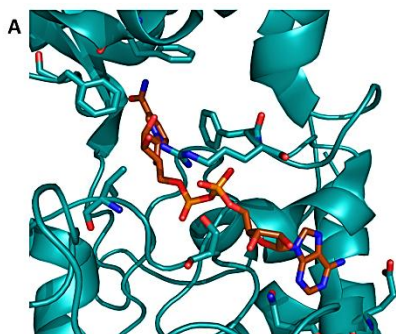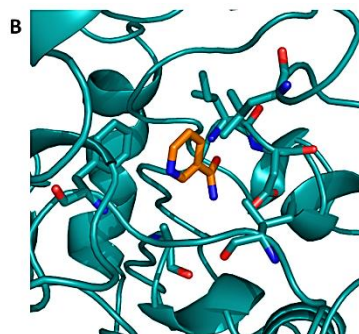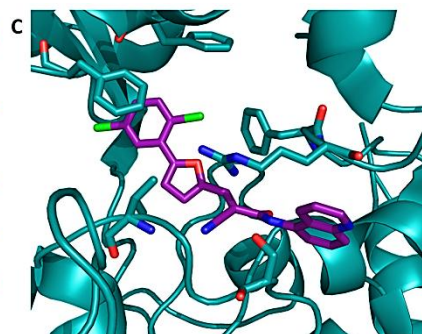

Supplement: Figure S3 — NAD+ (A), nicotinamide (B) and AGK2 (C) best-ranked docking poses in the hSIRT2 productive form. Protein is represented in deep cyan cartoon whereas the amino acids involved in the interaction with the ligands are represented as capped sticks. NAD+, Nicotinamide and AGK2 are also represented as capped sticks and are colored in brown, orange and purple, respectively. (PDF) [file pntd.0002689.s003.pdf]

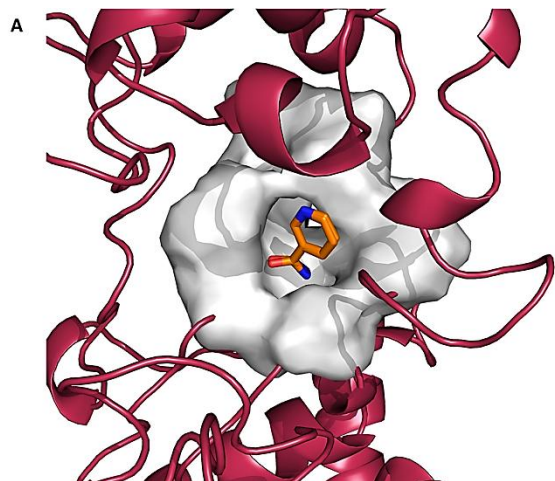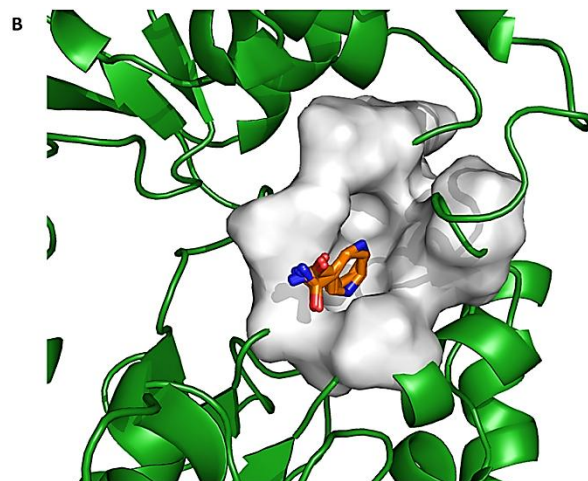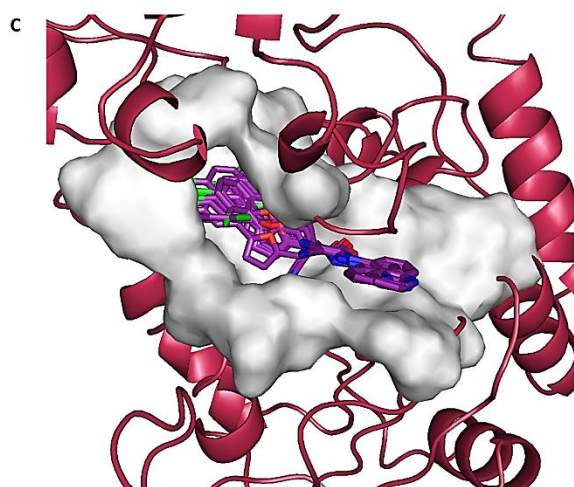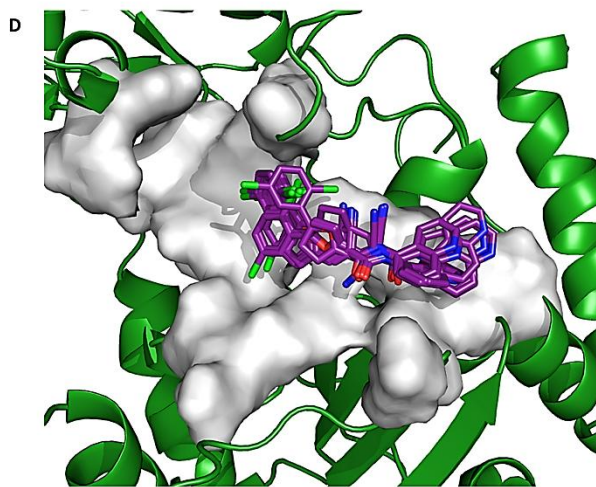

Supplement: Figure S4 — Superposition of ten nicotinamide and AGK2 docking poses in TcSIR2rp1 productive (A–C) and non-productive (B–D) forms. Nicotinamide and AGK2 are represented by orange and purple-capped sticks respectively. Backbones are represented with ribbons and are colored in dark pink and green, representing the TcSIR2rp1 productive and non-productive form respectively. Surfaces of the protein pockets are colored in gray. (PDF) [file pntd.0002689.s004.pdf]

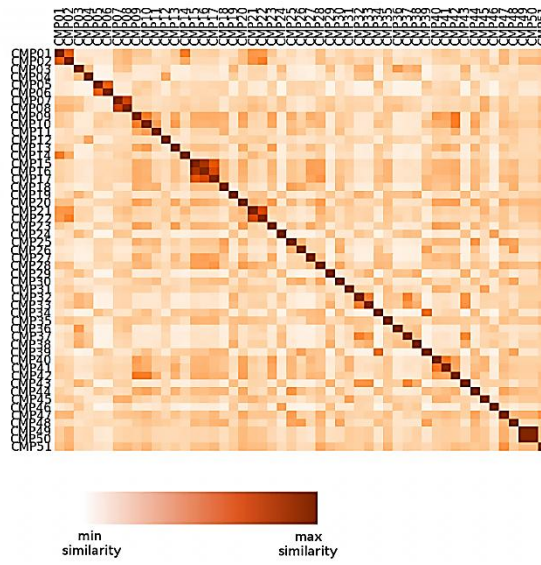

Supplement: Figure S5 — Heat map constructed from the fifty natural compounds of the library. Colors in the heat map indicate the relative similarity (brown for high similarity and white for low similarity) of the molecules in the dataset. (PDF) [file pntd.0002689.s005.pdf]

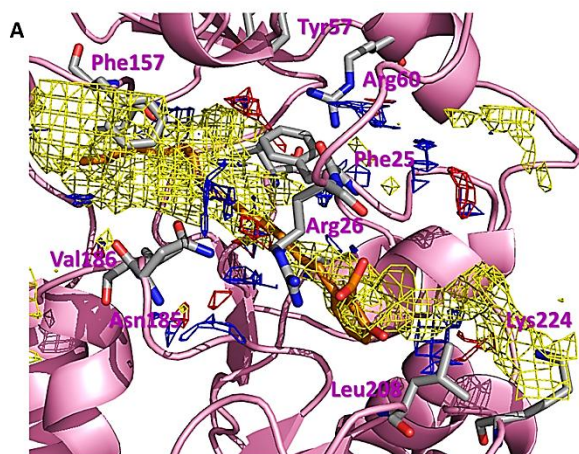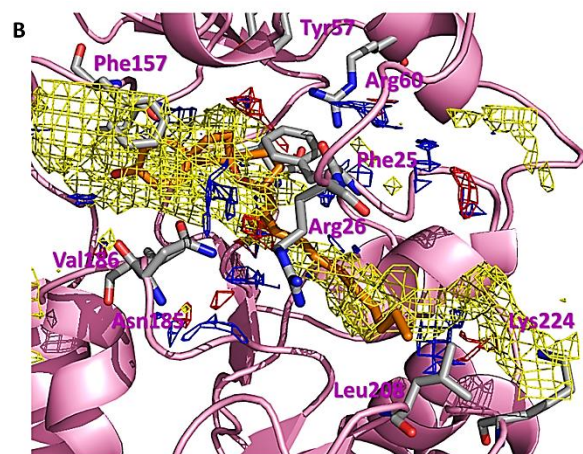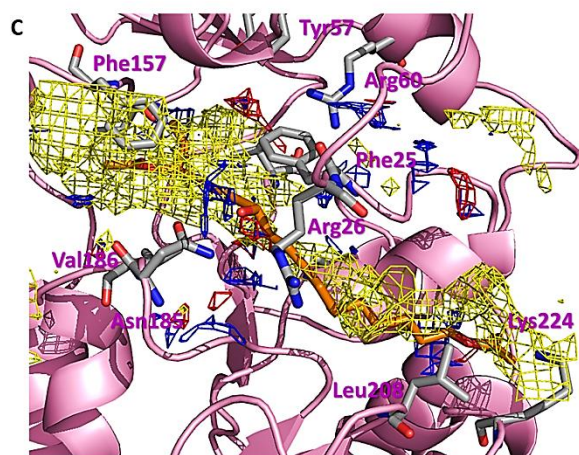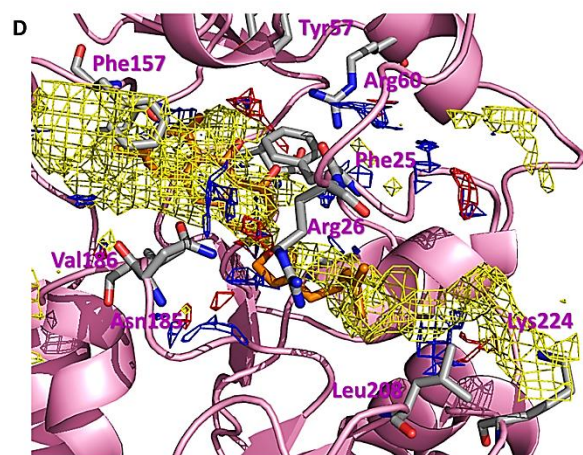

Supplement: Figure S6 — Molecular docking results in the productive pocket of TcSIR2rp3. (A) Anacardic acid docking pose in the TcSIR2rp3 productive form. (B) Aculeatin D best-ranked docking pose in the TcSIR2rp3 productive form. (C) 16-acetoxy-11-hydroxyoctadeca-17-ene-12,14-diynylethanoate best-ranked docking pose in the TcSIR2rp3 productive form. (D) Vismione D best-ranked docking pose in the TcSIR2rp3 productive form. Protein structures are represented as light pink ribbons. Amino acids participating in protein-ligand interactions are highlighted by light gray sticks. Ligands are represented as capped sticks and are colored in orange. GRID surface are also reported in the active site pocket and are colored yellow, blue and red for hydrophobic, electro-donor and electro-acceptor properties, respectively. (PDF) [file pntd.0002689.s006.pdf]

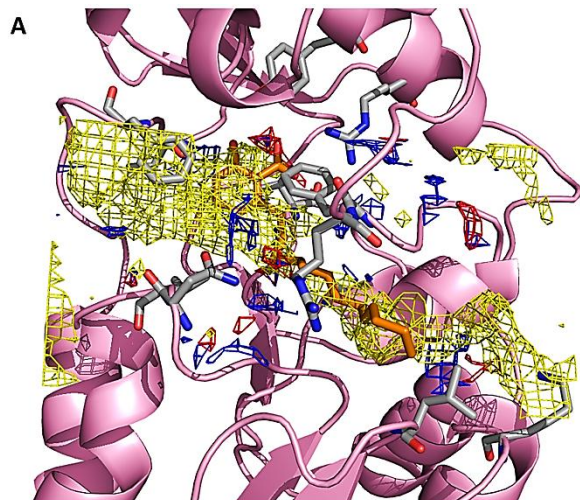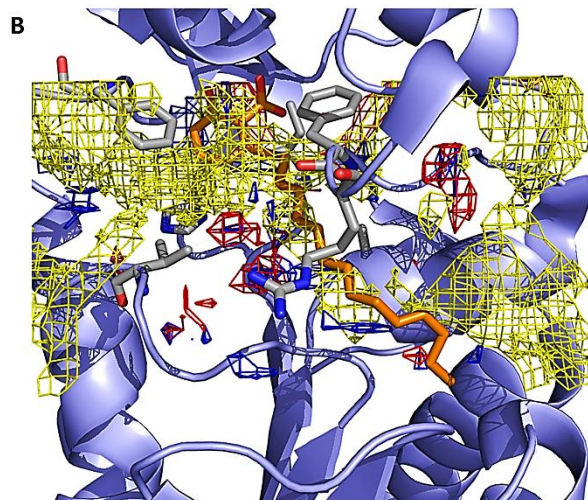

Supplement: Figure S7 — Best-ranked docking poses for the Anacardic acid derivative in TcSIR2rp3 (A) and hSIRT5 (B) productive forms. Protein structures are represented in ribbons and colored in pink and light blue for TcSIR2rp3 and hSIRT5, respectively. Amino acids participating in protein-ligand interactions are represented in orange capped sticks. Ligand is represented in capped stick and colored in orange. GRID surfaces are also reported in the active site pockets and colored as yellow, blue and red for highlighting hydrophobic, electro-donor and electro-acceptor properties, respectively. (PDF) [file pntd.0002689.s007.pdf]

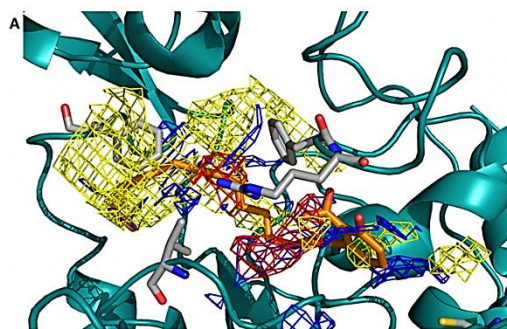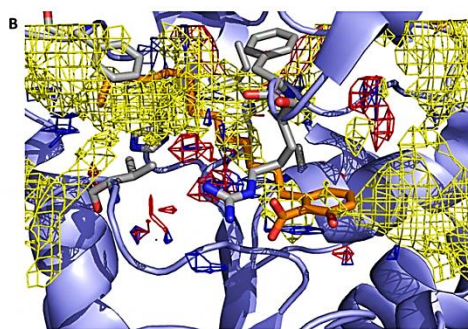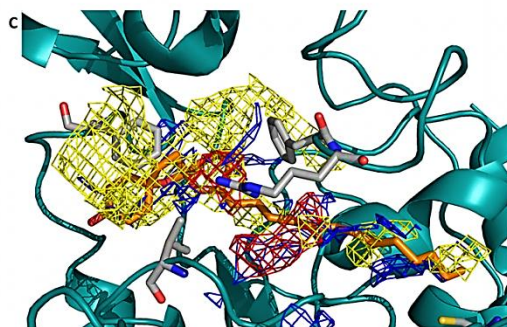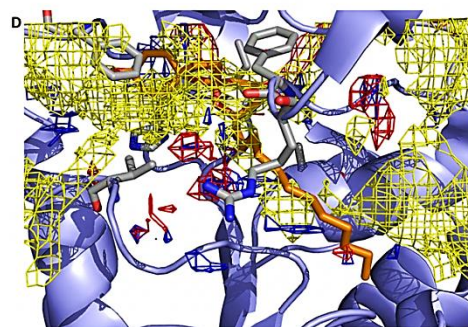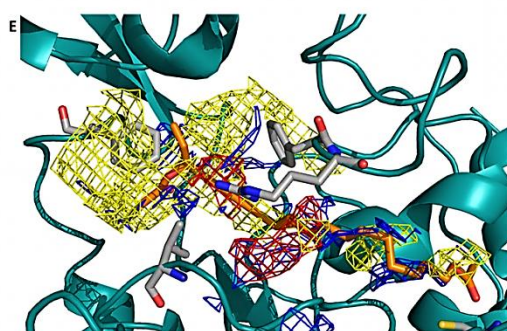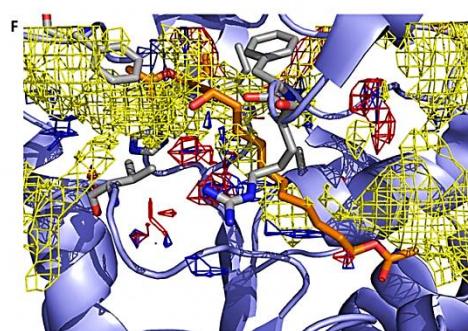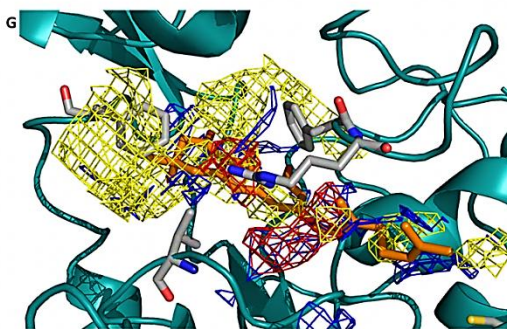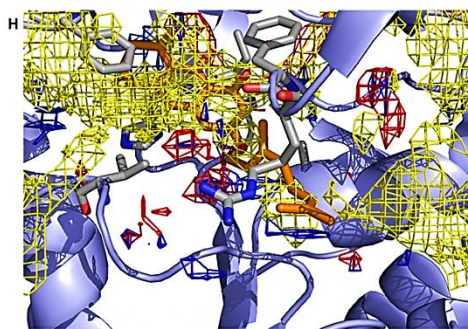

Supplement: Figure S8 — Molecular docking results from the virtual screening in the productive conformational states of hSIRT2 and hSIRT5. Anacardic acid best-ranked docking poses in hSIRT2 (A) and in hSIRT5 (B). Aculeatin D best-ranked docking poses in hSIRT2 (C) and in hSIRT5 (D). 16-acetoxy-11-hydroxyoctadeca-17-ene-12,14-diynylethanoate best-ranked docking poses in hSIRT2 (E) and in hSIRT5 (F). Vismione D best-ranked docking poses in hSIRT2 (G) and in hSIRT5 (H). Protein structures are represented as ribbons and are colored cyan and light blue for hSIRT2 and hSIRT5 respectively. Amino acids participating in protein-ligand interactions are represented as light gray sticks. Capped stick ligands are colored orange. GRID surfaces are also reported in the active site pockets and are colored yellow, blue and red to highlight hydrophobic, electron-donor and electron-acceptor properties, respectively. (PDF) [file pntd.0002689.s008.pdf]
