# Supplementary material for: Computational Studies on Sirtuins from Trypanosoma cruzi: Structures, Conformations and Interactions with Phytochemicals
Source: PLoS Negl Trop Dis. 2014 Feb 13;8(2):e2689. doi: 10.1371/journal.pntd.0002689 (PMC3923677; doi:10.1371/journal.pntd.0002689)
Supplement: Table S1 — Smile codes for the anti- T. cruzi compounds characterizing the natural product library. (PDF) [file pntd.0002689.s009.pdf]

N°	Chemical names	Smile Codes
1	Acetoxydihydroxyheptandecene	C=CC(C#CC#CC(O)CCCCCCCCCCCOC(C)=O)OC(C)=O
2	AcetylDglucopyranosyldiderroside	O=C(OC)C1=CO[C@@H](OC2C(O)C(O)C(O)C(COC(C)=O)O2)[C@@H]([C@@H](OC(C)=O)C)[C@@H]1C(O)=O
3	Ambigol A	ClC1=CC(Cl)=C(OC2=CC=C(Cl)C=C2Cl)C(O)=C1C3=CC(Cl)=CC(Cl)=C3O
4	Ambigol C	ClC1=C(O)C(Cl)=C(OC2=CC=C(Cl)C=C2Cl)C=C1OC3=CC(Cl)=CC(Cl)=C3
5	Anacardicacid (8E, 11E)	O=C(O)C1=C(CCCCCCC/C=C/C/C=C/CCC)C=CC=C1O
6	Ancistectorine A2	C[C@H]1C2=C(O)C=C(OC)C=C2C(C3=CC=C(OC)C4=C3C=CC=C4OC)[C@H](C)N1
7	Ancistrogriffine C	COC1=C(C2=CC=C(O)C3=C2C=C(C)C=C3OC)C(O)=C([C@H](C)N([H])[C@@H](C)C4)C4=C1
8	Ancistrogriffithine A	OC1=C(C2=CC(C3=CC(C4=C(OC)C([C@@H](C)N[C@@H](C)C5)=C5C=C4O)=C(C=C(C)C=C6OC)C6=C3O)=C(O)C7=C2C=C(C)C=C7OC)C(OC)=C([C@@H](C)N([H])[C@@H](C)C8)C8=C1
9	Angoroside C	OC1=CC=C(/C=C/C(O[C@@H]2[C@@H](CO[C@H]3[C@H](O)[C@@H](O)[C@@H](O)CO3)O[C@@H](OCCC4=CC(O)=C(OC)C=C4)[C@H](O)
10	Caaverine	[H][C@@]1(N([H])CC2)CC3=CC=CC=C3C4=C1C2=CC(OC)=C4O
11	Caffeicacid	OC1=C(O)C=C(/C=C/C(O)=O)C=C1
12	Chaetoxanthone A	COC1=C2C(OC(C=C(O[C@]3(C)O[C@H]4C[C@@H](O)C3)C4=C5O)=C5C2=O)=CC=C1
13	Chaetoxanthone B	COC1=C2C(OC(C=C(O[C@]3(C)O[C@H]4CCC3)C4=C5O)=C5C2=O)=CC=C1
14	Chaetoxanthone C	OC(C([C@]1([H])O[C@H](C)CCC1)=C2O)=CC3=C2C(C4=C(OC)C=CC=C4O3)=O
15	Cissampelofavone	O=C1C=C(C2=CC=C(O)C(OC)=C2)OC3=C1C=C(C(C(C4=C(O)C=C(OC)C=C4OC)=O)=C(C5=CC=C(OC)C=C5)O6)C6=C3
16	Deacetylgedunin	O=C1[C@@H]2[C@]3(O2)[C@]4(C)[C@H](O)CC5[C@](C)(C)C(C=C[C@]5(C)C4CC[C@@]3(C)[C@H](C6=COC=C6)O1)=O
17	DemethylpraecansonA	OC1=C(C=CC(C)(C)O2)C2=CC(OC)=C1C(/C([H])=C(O)/C3=CC=CC=C3)=O
18	2.2-dimethyl-6-carboxyethenyl-2H-1-benzopyrane	OC(/C=C/C1=CC=C(OC(C)(C)C=C2)C2=C1)=O
19	2.2-dimethyl-6-carboxyethenyl-8-prenyl2H-1-benzopyrane	OC(/C=C/C1=CC(C/C=C(C)/C)=C(OC(C)(C)C=C2)C2=C1)=O
20	Dimethylmethoxybenzochromenylmethanol	COC1=CC2=C(C=C1)OC(C)(C)C3=CC=C(CO)C=C32
21	Entkaurenoicacid	C[C@]12C(CC[C@]3(CC4=C)C2CC[C@@H]4C3)[C@@](CC(O)=O)(C)CCC1
22	EntnaringeninylOmethylnaringeninyl	OC1=CC(O)=C(C([C@@H](C2=C3C(C(C[C@@H](C4=CC=C(OC)C=C4)O3)=O)=C(O)C=C2O)[C@H](C5=CC=C(O)C=C5)O6)=O)C6=C1
23	Gallocatechindigallate	OC(C(OC(C1=CC(O)=C(O)C(O)=C1)=O)=C2)=C(O)C=C2C3C(OC(C4=CC(O)=C(O)C(O)=C4)=O)C(C5=C(O)C=C(O)C=C5O3)=O
24	Gamafagarine	COC1=C2C(OC=C2)=NC3=C(OC)C=CC=C31
25	Garciaxanthone B	C=CC(C)(C)C1=C(O)C2=C(C(O)=C1)OC3=C4C(C=CC(C)(C)O4)=CC=C3C2=O
26	Geranylgeraniol	C/C(C)=C/CC/C(C)=C/CC/C(C)=C/CC/C(C)=C/CO
27	Gravicin A	O=C1C2=CC=CC(O)=C2OC3=C1C(O)=CC(/C=C/[C@@]4(C)[C@](C5=CC(O)=C(C(C(C=CC=C6O)=C6O7)=O)C7=C5O)([H])C=C(C)CC4)=C3O
28	Haemanthamine	[H][C@@]12N3CC4=CC(OCO5)=C5C=C4[C@@]1([C@@H](O)C3)C=CC(OC)C2
29	Helenalin	O=C1C=C[C@]2([H])[C@]1(C)[C@@H](O)[C@H](C(C(O3)=O)=C)[C@H]3C[C@H]2C
30	hydroxyanthecotulide	C/C(C)=C\C(C/C(C)=C/[C@H](O)[C@@H]1COC(C1=C)=O)=O
31	Hydroxydaidzein	OC1=C2C(C(C(C3=CC=C(O)C(O)=C3)=CO2)=O)=CC=C1
32	Hydroxymethylbutendimethoxytetrahydrofuroquinoliylacetate	COC1=C2C(OC=C2)=NC3=C1CCC(OC(C)=O)[C@]3(/C=C/C(C)(O)C)OC
33	Ioxadispiroketalaculeatin D	O[C@H](C1)C[C@@H](CCCCCCCCCCCCC)O[C@]21CC[C@]3(C=CC(C=C3)=O)O2
34	Ivalin	O[C@H]1CC([C@@](C[C@H](C(C(O2)=O)=C)[C@H]2C3)([H])[C@]3(C)C1)=C
35	Komorovinone	C[C@@]1(C)CCC[C@@]2(O3)C1C[C@@]3(O)C(C(C(C(C)C)=C(OC)C4=O)=O)=C4C2
36	Luteolin	O=C1C=C(C2=CC=C(O)C(O)=C2)OC3=C1C(O)=CC(O)=C3
37	Machilin G	C[C@H]1[C@@H](C)[C@H](C2=CC=C(OC)C(OC)=C2)O[C@@H]1C3=CC(OCO4)=C4C=C3
38	Methylpluviatolide	O=C1OC[C@H](CC2=CC=C3C(OCO3)=C2)[C@H]1CC4=CC(OC)=C(O)C=C4
39	OdimethylancistrocladiniumA	COC1=CC(OC)=C(C(C)=[N+](C2=C(C=C(C)C=C3O)C3=C(OC)C=C2)[C@@H](C)C4)C4=C1

**40**	Parthenolide	C/C1=C\CC[C@]2(C)[C@@H](O2)[C@@H](OC(C3=C)=O)[C@H]3CC1
**41**	Phenylanthraquinonesknipholone	O=C1C2=C(C(C3=C(O)C(C(C)=O)=C(OC)C=C3O)=C(C)C=C2O)C(C4=CC=CC(O)=C41)=O
**42**	Piperine	O=C(N1CCCCC1)/C=C/C=C/C2=CC(OCO3)=C3C=C2
**43**	3-Prenyl-4-hydroxycinnamicacid	OC(/C=C/C1=CC=C(O)C(C/C=C(C)/C)=C1)=O
**44**	3.5-Prenyl-4-hydroxycinnamicacid	OC(/C=C/C1=CC(C/C=C(C)/C)=C(O)C(C/C=C(C)/C)=C1)=O
**45**	Sarachine	C[C@@H](C1([H])CC[C@H](C)CN1[H])[C@@]2([H])CCC3C4CC=C5C[C@@H](N)CC[C@]5(C)C4CC[C@@]32C
**46**	Sophoraflavone G	OC1=CC(O)=C(C(C[C@@H](C2=C(O)C=C(O)C=C2)O3)=O)C3=C1CC(C(C)=C)C/C=C(C)\C
**47**	Tiliroside	O=C1C(O[C@@H]2O[C@H](CCC(/C=C/C3=CC=C(O)C=C3)=O)[C@@H](O)[C@H](O)[C@H]2O)=C(C4=CC=C(O)C=C4)OC5=C1C(O)=CC(O)=C5
**48**	Usnicacid R	O=C1C(C(C)=O)C([C@]2(C)C3=C(O)C(C)=C(O)C(C(C)=O)=C3OC2=C1)=O
**49**	Usnicacid S	OC1=C(C)C(O)=C2C(OC(C2(C)C3=O)=CC(C3C(C)=O)=O)=C1C(C)=O
**50**	Vismione D	C\C(C)=C/CC/C(C)=C/COC(C=C1O)=CC(C1=C2O)=CC3=C2C(CC(O)(C)C3)=O
