# Supplementary material for: Computational Studies on Sirtuins from Trypanosoma cruzi: Structures, Conformations and Interactions with Phytochemicals
Source: PLoS Negl Trop Dis. 2014 Feb 13;8(2):e2689. doi: 10.1371/journal.pntd.0002689 (PMC3923677; doi:10.1371/journal.pntd.0002689)
Supplement: Table S2 — Summary of the residues characterizing the A–C pockets of TcSir2rp1 and hSIRT2. (PDF) [file pntd.0002689.s010.pdf]

	TcSir2rp1	hSIRT2
**Pocket A**	A38, G39, V42, A 43, G213, N238, L239, D307, C308	A85, G86, T89, S90, G261, N286, K287, E323, C324
**Pocket B**	F49, R50, I 56, P68, Q122, H142, V218	F96, R97, L103, P115, Q167, H187, V266
**Pocket C**	S41, H104, T121, N123, I124, D125	S88, H149, T166, N168, I169, D170
