# Supplementary material for: Computational Studies on Sirtuins from Trypanosoma cruzi: Structures, Conformations and Interactions with Phytochemicals
Source: PLoS Negl Trop Dis. 2014 Feb 13;8(2):e2689. doi: 10.1371/journal.pntd.0002689 (PMC3923677; doi:10.1371/journal.pntd.0002689)
Supplement: Table S3 — Best-ranked PLP docking scores of NAD+, nicotinamide and AGK2 ligands in the non-productive/productive TcSIR2rp1 conformations. (PDF) [file pntd.0002689.s011.pdf]

	TcSir2rp1 non-productive conformation	Tcsir2rp1 productive conformation
**NAD^+**	51.6	94.3
**Nicotinamide**	28.3	34.6
**AGK2**	58.3	71.2
