# Supplementary material for: Computational Studies on Sirtuins from Trypanosoma cruzi: Structures, Conformations and Interactions with Phytochemicals
Source: PLoS Negl Trop Dis. 2014 Feb 13;8(2):e2689. doi: 10.1371/journal.pntd.0002689 (PMC3923677; doi:10.1371/journal.pntd.0002689)
Supplement: Table S4 — Clustering analysis results (2 Å cut-off). Red clusters contain the best-ranked poses according to the PLP score. (PDF) [file pntd.0002689.s012.pdf]

| | Non-productive TcSIR2rp1 | | | Productive TcSIR2rp1 | | |
|---|---|---|---|---|---|---|
| | NAD | Nicotinamide | AGK2 | NAD | Nicotinamide | AGK2 |
| Cluster 1 | 24 | 99 | 16 | 46 | 100 | 28 |
| Cluster 2 | 1 | 1 | 5 | 1 | | 3 |
| Cluster 3 | 1 | | 15 | 17 | | 5 |
| Cluster 4 | 6 | | 45 | 2 | | 7 |
| Cluster 5 | 12 | | 14 | 3 | | 17 |
| Cluster 6 | 1 | | 5 | 1 | | 27 |
| Cluster 7 | 3 | | | 2 | | 10 |
| Cluster 8 | 7 | | | 5 | | 1 |
| Cluster 9 | 1 | | | 4 | | 4 |
| Cluster 10 | 4 | | | 2 | | |
| Cluster 11 | 6 | | | 2 | | |
| Cluster 12 | 5 | | | 1 | | |
| Cluster 13 | 1 | | | 2 | | |
| Cluster 14 | 2 | | | 1 | | |
| Cluster 15 | 12 | | | 1 | | |
| Cluster 16 | 1 | | | 1 | | |
| Cluster 17 | 2 | | | 2 | | |
| Cluster 18 | 1 | | | 3 | | |
| Cluster 19 | 1 | | | 3 | | |
| Cluster 20 | 1 | | | 1 | | |
| Cluster 21 | 3 | | | | | |
| Cluster 22 | 1 | | | | | |
| Cluster 23 | 1 | | | | | |
| Cluster 24 | 3 | | | | | |
